# Comparison of Two Human Skin Cell Isolation Protocols and Their Influence on Keratinocyte and Fibroblast Culture

**DOI:** 10.3390/ijms241914712

**Published:** 2023-09-28

**Authors:** Álvaro Sierra-Sánchez, Martin A. Barbier, Brice Magne, Danielle Larouche, Salvador Arias-Santiago, Lucie Germain

**Affiliations:** 1 LOEX Tissue Engineering Laboratory, Université Laval Research Center and Department of Surgery, Faculty of Medicine, Université Laval, Québec City, QC G1J 1Z4, Canada; alvarosisan@gmail.com (Á.S.-S.); martin.barbier@crchudequebec.ulaval.ca (M.A.B.); magneb@chop.edu (B.M.); danielle.larouche@crchudequebec.ulaval.ca (D.L.); lucie.germain@fmed.ulaval.ca (L.G.); 2 Division of Regenerative Medicine, CHU de Québec—Université Laval Research Center, Québec City, QC G1J 1Z4, Canada; 3 Unidad de Producción Celular e Ingeniería Tisular (UPCIT), Virgen de las Nieves University Hospital, ibs.Granada, Andalusian Network of Design and Translation of Advanced Therapies, 18014 Granada, Spain; 4 Department of Dermatology, Virgen de las Nieves University Hospital, 18012 Granada, Spain; 5 Department of Dermatology, Faculty of Medicine, University of Granada, 18016 Granada, Spain

**Keywords:** skin cell isolation, CFE, fibroblast, Keratin 19, keratinocyte, protocol, skin substitute, tissue engineering

## Abstract

For the development of advanced therapies, the use of primary cells instead of cell lines is preferred. The manufacture of human tissue-engineered skin substitutes requires efficient isolation and culture protocols allowing a massive expansion of the cells in culture from an initial specimen of a minimal size. This study compared two skin cell isolation protocols, routinely applied in two clinical laboratories. Epithelial (keratinocytes) and dermal (fibroblasts) cells were isolated and cultured from three human skin biopsies (N = 3). The two-step digestion protocol (LOEX-Protocol) firstly used thermolysin to enzymatically disrupt the dermal–epidermal junction while, for the one-step digestion protocol (UPCIT-Protocol), mechanical detachment with scissors was applied. Then, the epidermal and dermal layers were digested, respectively, to achieve cell isolation. The cell size, viability, yield and growth were analyzed over five passages (P). The colony-forming efficiency (CFE) and Keratin 19 (K19) expression of epithelial cells were also assessed after P0 and P1. Regarding the dermal cells, no significant differences were observed in the tested parameters of isolation and culture. However, for the epithelial cells, viability was higher (93% vs. 85%) and the number of cells extracted per cm^2^ of skin was 3.4 times higher using the LOEX-Protocol compared to the UPCIT-Protocol. No significant difference was observed for any parameter once the keratinocytes were cultured from P1 to P4. The CFE and K19 expression decreased from P0 to P1 in both protocols, probably due to the culture process. This study shows that both protocols enable the efficient isolation of skin dermal and epithelial cells and subsequent culture to produce grafts destined for the treatment of patients.

## 1. Introduction

Immortalized or commercial cell lines are widely used in research due to their cell growth potential and well-known characteristics [1]. However, primary human cells are valuable in biomedical sciences for evaluating or characterizing different drugs, substances or molecules, for example [2]. Moreover, as advanced therapies are developing, human cells are used alone or combined with biomaterials and/or devices for the treatment of patients [3]. For this clinical purpose, the control of experimental and manufacturing conditions for autologous primary cell cultures from a tissue biopsy is a key step [2].

To establish a primary cell culture, different aspects such as the cell isolation protocol, cell attachment to plastic surfaces and culture media must be considered [1]. To initiate a primary culture from a biopsy, tissue explants can be seeded on culture flasks allowing cells to migrate out and proliferate. Alternatively, cells can be isolated by disaggregating the tissue mechanically and/or enzymatically to produce a suspension of cells [4] that will be seeded on flasks and cultured using the appropriate growth medium.

Therefore, it is important to optimize and use a successful isolation protocol that preserves the properties and characteristics of the different native human cell types as much as possible. Moreover, these methods should be robust, standardized and report a good cell yield to be able to reduce the manufacturing time in the case where the cell/tissue grafts are required to treat patients [5,6]. 

Manufacturing tissue-engineered skin substitutes (TESSs) for clinical purposes, such as burn treatment, requires the use of autologous skin cells, commonly keratinocytes and fibroblasts, the main cell types of the epidermis/appendages and the dermis, respectively [7]. For this reason, and the limited size of the donor skin that is usually available for cell extraction, it is important to have an effective isolation protocol to be able to culture and expand those cells without affecting their properties.

Despite the similarities between most skin cell isolation procedures, different protocols exist depending on the number and type of enzymatic digestions or the addition of mechanical disaggregation or not [8]. Among them, the LOEX Tissue Engineering Laboratory (Canada) [9,10] applies a two-step digestion protocol in which human skin samples are enzymatically digested (first digestion step) to facilitate the mechanical detachment of the epithelium (epidermis and hair follicles) from the dermis. The next day, the epidermis and the dermis are separately incubated with specific enzymes to obtain the primary keratinocytes and fibroblasts, respectively (second digestion step). In contrast, the protocol applied at the Unidad de Producción Celular e Ingeniería Tisular (Spain) [11], is based on a mechanical removal of the dermis from the epidermis with scissors. Then, epithelial cells are enzymatically disaggregated, plated and cultured, while the dermis is digested for 16–20 h resulting in fibroblast isolation and culture the following day (single digestion step). 

**Figure 1:**
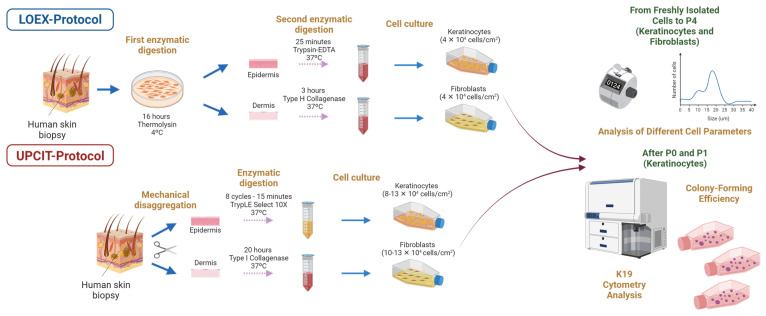
Schematic representation of the research study. Created with BioRender.com.

The aim of this study was to compare these two isolation protocols in terms of the cell yield and size, cell proliferation and population doubling time of keratinocytes and fibroblasts over five passages (P0 to P4). The isolation protocols compared were as follows: (i) the one applied at the LOEX Tissue Engineering Laboratory (LOEX-Protocol) and (ii) the one used at the Unidad de Producción Celular e Ingeniería Tisular (UPCIT-Protocol). Moreover, the presence of proliferative and stem cells in the cultures of keratinocytes was evaluated by colony-forming efficiency (CFE) assays and flow cytometry analysis during the primary culture and first passage (Figure 1).

## 2. Results

### 2.1. Cell Size, Viability and Yield

#### 2.1.1. Epithelial Cells

**Figure 2:**
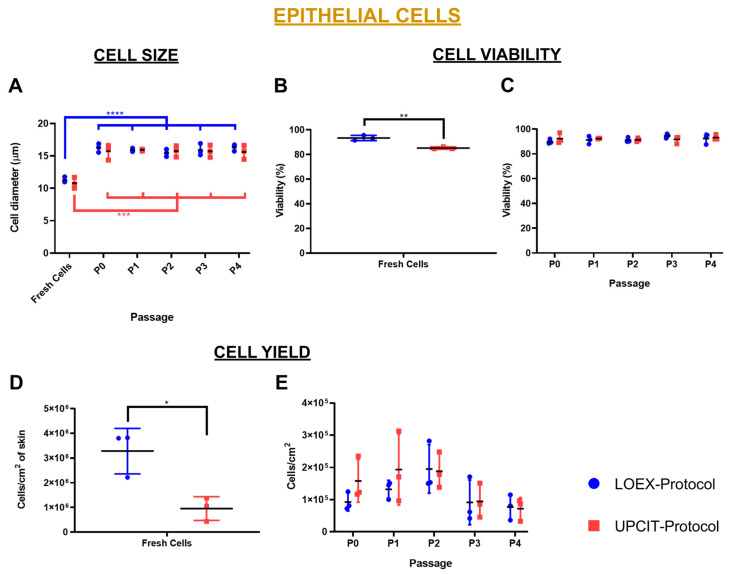
Comparison of cell size, viability and yield of epithelial cells/keratinocytes. (**A**) Cell size (Ø); (**B**) cell viability after cell isolation from the biopsy (Fresh Cells); (**C**) cell viability after each passage (P0–P4); (**D**) number of cells isolated per cm^2^ of skin; (**E**) number of cells recovered after each passage per culture surface area (P0–P4). Statistical significance: * *p*-value < 0.5; ** *p*-value < 0.01; *** *p*-value < 0.001; **** *p*-value < 0.0001 (one-way ANOVA and Tukey’s multiple comparisons tests for comparison between passages for each protocol and multiple *t*-tests and Holm–Sidak method for comparison between protocols for each passage). Data are shown as mean value (horizontal black line) ± SD (N = 3 for each protocol (the individual values are also represented)). LOEX-Protocol: two-step digestion protocol; UPCIT-Protocol: one-step digestion protocol; P: passage; P0: primary culture.

The size (diameter, Ø) of the epithelial cells isolated with both the LOEX- and UPCIT-Protocols was similar at each time point (freshly isolated and at each passage). For both methods, the freshly isolated cells were significantly (*p*-value < 0.001) smaller than the cultured keratinocytes (P0 to P4) (Figure 2A).

The evaluation of cell viability indicated that the viability of freshly isolated epithelial cells was higher when the LOEX-Protocol was used compared to the UPCIT-Protocol (93 ± 2% vs. 85 ± 1%, respectively; *p*-value < 0.01). However, after cell culture over several passages, no significant differences were observed. The cell viability varied between 89% and 95% for the cultured keratinocytes (P0 to P4) for both protocols (Figure 2B,C). 

The cell yield after isolation from the skin biopsy was significantly higher (*p*-value < 0.05) with the LOEX-Protocol (3.28 × 10^6^ ± 0.92 × 10^6^ cells/cm^2^ biopsy) than with the UPCIT-Protocol (9.52 × 10^5^ ± 4.81 × 10^5^ cells/cm^2^ biopsy) (Figure 2D). No significant differences were observed between the protocols in the number of keratinocytes recovered per cm^2^ for a given passage between P0 and P4 (Figure 2E). 

#### 2.1.2. Dermal Cells

**Figure 3:**
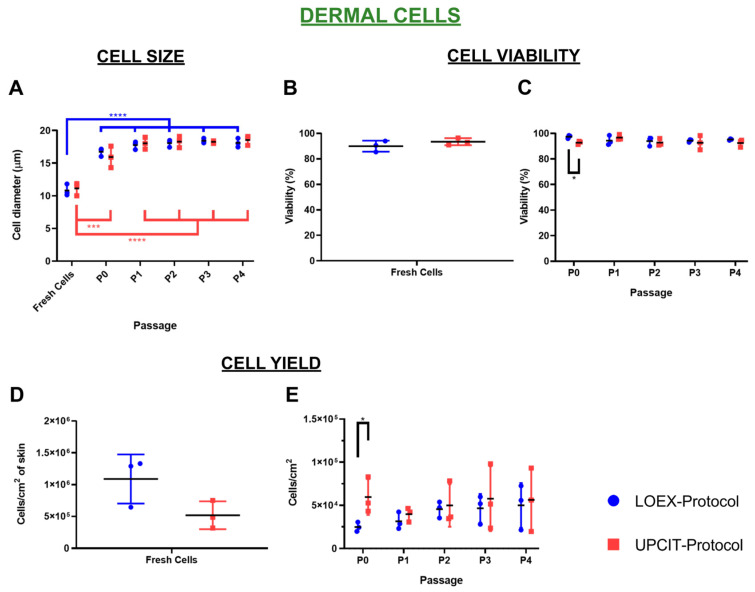
Comparison of cell size, viability and yield of dermal cells/fibroblasts. (**A**) Cell size (Ø); (**B**) cell viability after cell isolation from the biopsy (Fresh Cells); (**C**) cell viability after each passage (P0–P4); (**D**) number of cells isolated per cm^2^ of skin; (**E**) number of cells recovered after each passage per culture surface area (P0–P4). Note that 2.8 times more freshly isolated dermal cells were seeded by culture surface area in the UPCIT-Protocol compared to the LOEX-Protocol. Statistical significance: * *p*-value < 0.5; *** *p*-value < 0.001; **** *p*-value < 0.0001 (one-way ANOVA and Tukey’s multiple comparisons tests for comparison between passages for each protocol and multiple *t*-tests and Holm–Sidak method for comparison between protocols for each passage). Data are shown as mean value (horizontal black line) ± SD (N = 3 for each protocol (the individual values are also represented)). LOEX-Protocol: two-step digestion protocol; UPCIT-Protocol: one-step digestion protocol; P: passage; P0: primary culture.

As for the epithelial cells, the size of the dermal cells was significantly lower just after isolation compared to the cultured cells (P0 to P4) for the LOEX- and UPCIT-Protocols (*p*-value < 0.001 and *p*-value < 0.0001). No significant difference was observed between the protocols at each time point (fresh cells, P0 to P4) (Figure 3A).

The cell viability of the freshly isolated cells (Figure 3B) and cultured cells was over 90% using both protocols, although small differences were observed after P0 (*p*-value < 0.05; Figure 3C).

Regarding cell yield, although a higher number of freshly isolated dermal cells was obtained using the LOEX-Protocol compared to the UPCIT-Protocol, no significant difference was reported (Figure 3D). Although more fibroblasts were recovered after P0 with the UPCIT-Protocol compared to the LOEX-Protocol (2.4-fold higher, *p*-value < 0.05; Figure 3E), it must be stated that the initial number of freshly isolated dermal cells seeded by culture surface area was also higher (2.8 times). 

### 2.2. Culture and Population Doubling Time

#### 2.2.1. Epithelial Cells

**Figure 4:**
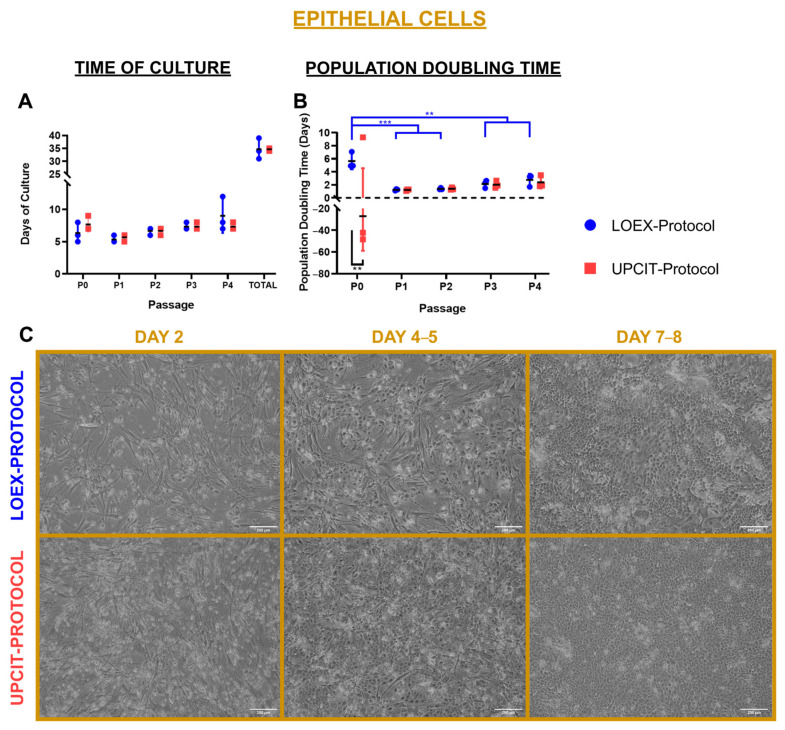
Comparison of culture time and population doubling time of epithelial cells/keratinocytes. (**A**) Time to reach confluence for different passages; (**B**) population doubling time; (**C**) representative pictures of keratinocytes’ culture after 2, 4–5, 7–8 days of primary culture. Scale bar: 200 µm. Statistical significance: ** *p*-value < 0.01; *** *p*-value < 0.001 (one-way ANOVA and Tukey’s multiple comparisons tests for comparison between passages for each protocol and multiple *t*-tests and Holm–Sidak method for comparison between protocols for each passage). Data are shown as mean value (horizontal black line) ± SD (N = 3 for each protocol (the individual values are also represented)). LOEX-Protocol: two-step digestion protocol; UPCIT-Protocol: one-step digestion protocol; P: passage; P0: primary culture.

For keratinocyte cultures, the time necessary to reach confluence was similar for both protocols at each passage (Figure 4A). Moreover, a similar population doubling time was observed for both protocols at passages P1 to P4. A significant difference between the keratinocytes isolated by the two protocols was only observed in the primary culture (P0) (*p*-value < 0.01) (Figure 4B). The negative value for the UPCIT-Protocol (−27 ± 32 days) indicated that the initial number of epithelial cells seeded was higher than the number of keratinocytes recovered at the end of the culture period, although a high variability between skin biopsies was noticed (Figure 4B). For cells isolated with the LOEX-Protocol, the comparison of the population doubling time revealed significant differences between P0 and the other passages (*p*-value < 0.001 for P1 and P2; *p*-value < 0.01 for P3 and P4) (Figure 4B). Representative pictures of the keratinocytes cultured at P0 at various times are included in Figure 4C.

#### 2.2.2. Dermal Cells

**Figure 5:**
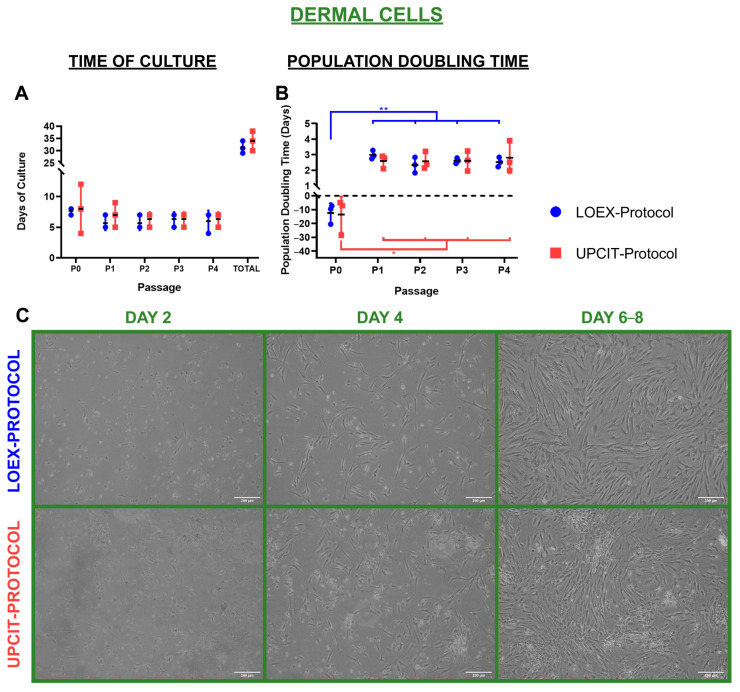
Comparison of culture and population doubling time of dermal cells/fibroblasts. (**A**) Time to reach confluence for different passages; (**B**) population doubling time; (**C**) representative pictures of fibroblasts’ culture after 2, 4, 6–8 days of primary culture. Scale bar: 200 µm. Statistical significance: * *p*-value < 0.05; ** *p*-value < 0.01 (one-way ANOVA and Tukey’s multiple comparisons tests for comparison between passages for each protocol and multiple *t*-tests and Holm–Sidak method for comparison between protocols for each passage). Data are shown as mean value (horizontal black line) ± SD (N = 3 for each protocol (the individual values are also represented)). LOEX-Protocol: two-step digestion protocol; UPCIT-Protocol: one-step digestion protocol; P: passage; P0: primary culture.

Fibroblasts isolated with the two protocols proliferated in culture and reached confluence at the same speed (Figure 5A), and their population doubling time was comparable (Figure 5B). However, for each method, a significant difference in the population doubling time between the primary culture (P0) and passages (P1 to P4) was observed (*p*-value < 0.01 for the LOEX-Protocol and *p*-value < 0.05 for the UPCIT-Protocol) (Figure 5B). The phenotype of the fibroblasts was similar (Figure 5C).

### 2.3. Clonogenicity of Epithelial Cell Cultures

Epithelial stem cells are known to be clonogenic. To evaluate the clonogenicity of the epithelial cells isolated from both protocols, the size and number of colonies formed by the keratinocytes were monitored. 

**Figure 6:**
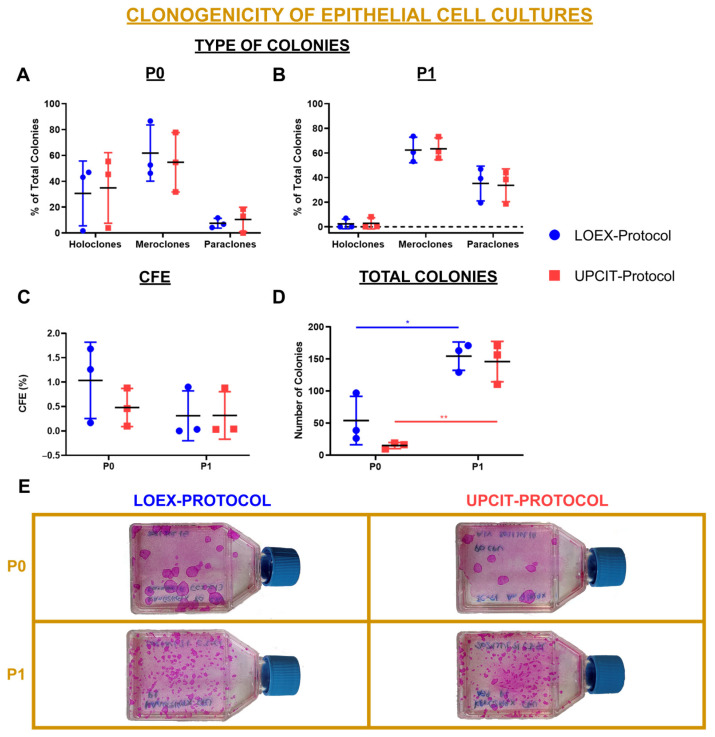
Clonogenicity of epithelial cells. (**A**) Comparison between protocols of the percentage of colonies that are holoclones, meroclones or paraclones after primary culture; (**B**) comparison between protocols of the percentage of colonies that are holoclones, meroclones or paraclones after P1; (**C**) colony-forming efficiency (CFE; the percentage of holoclones from the initial number of epithelial cells seeded); (**D**) total number of colonies; (**E**) macroscopic pictures of CFE assay after P0 and P1. Statistical significance: * *p*-value < 0.05; ** *p*-value < 0.01; for type of colony analysis: multiple *t*-tests and Holm–Sidak method for comparison between protocols for each type of colony and passage; for CFE and total colonies analysis: multiple *t*-tests and Holm–Sidak method for comparison between passages for each protocol and for comparison between protocols for each passage. Data are shown as mean value (horizontal black line) ± SD (N = 3 for each protocol (the individual values are also represented)). LOEX-Protocol: two-step digestion protocol; UPCIT-Protocol: one-step digestion protocol; P: passage; P0: primary culture.

Since the size of a colony reveals the proliferation potential of the epithelial cell that generated it, the diameter (Ø) of the colonies was measured to determine the percentage of holoclones (Ø ≥ 4.5 mm), meroclones (4.5 mm > Ø > 1.5 mm) and paraclones (Ø ≤ 1.5 mm). The proportion of holoclones, meroclones and paraclones was similar regardless of the cell isolation protocol applied (Figure 6A,B). Between P0 and P1, the percentage of holoclones decreased, whereas the proportion of meroclones and paraclones increased for both protocols.

A similar colony-forming efficiency (CFE)—defined as the percentage of holoclones from the initial number of epithelial cells seeded—was observed for both the LOEX- and UPCIT-Protocols but decreased from P0 to P1 (Figure 6C). However, the total number of colonies was higher after P1 compared to P0 in both protocols (*p*-value < 0.05 for the LOEX-Protocol and *p*-value < 0.01 for the UPCIT-Protocol), although no significant difference was observed regarding the isolation protocol used (Figure 6D). Representative pictures of CFE staining are included in Figure 6E.

### 2.4. Keratin 19 (K19) Analysis by Flow Cytometry

**Figure 7:**
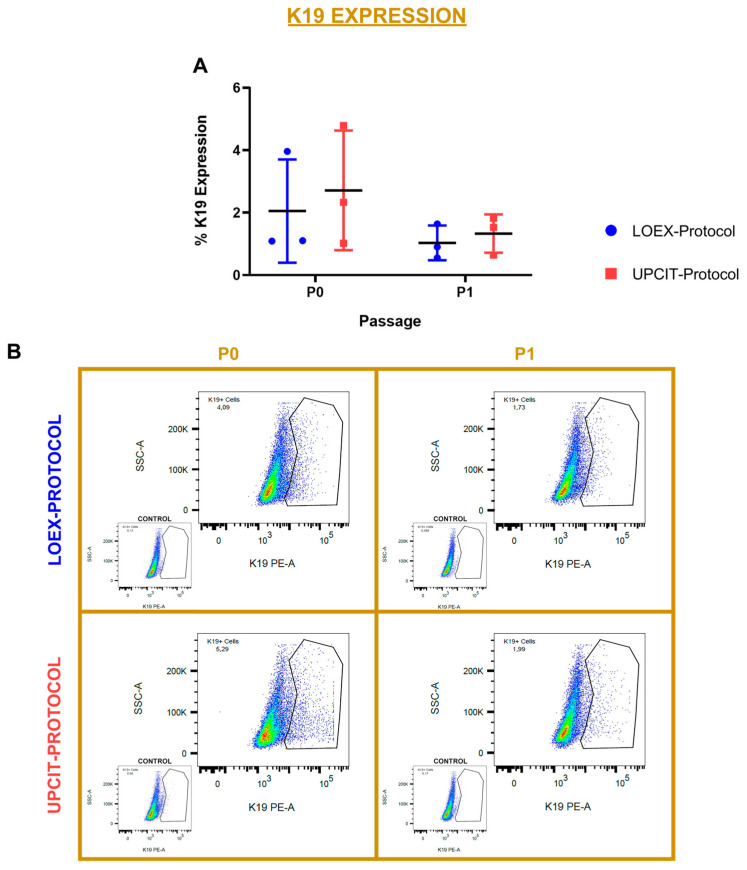
Comparison of Keratin 19 (K19) expression by flow cytometry. (**A**) Comparison between protocols of the percentage of K19^+^ epithelial cells after P0 and P1; (**B**) representative graphs of cytometry assays, including their controls. Multiple *t*-tests and Holm–Sidak method for comparison between passages for each protocol and for comparison between protocols for each passage. Data are shown as mean value (horizontal black line) ± SD (N = 3 for each protocol (the individual values are also represented)). LOEX-Protocol: two-step digestion protocol; UPCIT-Protocol: one-step digestion protocol; P: passage; P0: primary culture.

K19 is a marker associated with skin epithelial stem cells [12]. The proportion of keratinocytes expressing K19 was measured by flow cytometry at P0 and P1. The results indicated that no significant differences were observed regardless of the cell isolation protocol or the cell passage (Figure 7A). Representative pictures of the K19 cytometry analysis are included in Figure 7B.

## 3. Discussion

In this study, we compared two human skin cell isolation protocols that are used to manufacture human bilayered tissue-engineered skin substitutes for the treatment of severely burned patients. Each of these protocols was developed independently at the LOEX (Canada) [9,10] and the UPCIT (Spain) [11]. The side-by-side comparison of the optimized protocols has shown that the number of epithelial cells obtained per surface area of the same skin biopsies and their viability were higher with the LOEX-Protocol (Figure 2 and Figure 4). However, similar results were measured for the other evaluated parameters, such as clonogenicity and the number of colonies. For the dermal cells, no difference was observed between cell viability and yield following isolation, and the subsequent cultures exhibited the same behavior (Figure 3 and Figure 5). 

The main difference between both protocols is the first step, which is a mechanical preparation of the skin or an added digestion step. The protocols involving only one digestion step have been described, in which skin biopsies (epidermis–dermis) are incubated with a trypsin enzyme [13,14,15,16] to achieve epithelial cell isolation. However, the presence of the dermis results in the liberation of the fibroblasts that contaminate the epithelial cell population. To overcome this problem, other protocols, such as the UPCIT-Protocol [11], require mechanical isolation of the epidermis from the dermis with scissors to remove as much dermal tissue as possible [11] or other tools, such as foot planers, to obtain epidermal sheets that were subsequently digested [17]. The remaining dermis can be used for fibroblast isolation. 

Compared to one-step digestion protocols, the two-step digestion protocols are preferred mainly for epithelial cell isolation [18], and dispase [19,20,21,22] or thermolysin [9,10,23,24] are the two enzymes frequently used in this phase. In the case of the LOEX-Protocol, the added first digestion step with thermolysin allows the detachment of the epidermis from the dermis due to its specific effect between the bullous pemphigoid, located at the lamina lucida of the epithelial side [25], and the laminin of the dermal compartment, disrupting the hemidesmosomes more easily but preserving the desmosomes between keratinocytes [26]. This avoids the experience-based considerations of the researchers to decide when most of the dermis tissue is removed, as in the case of the UPCIT-Protocol.

For dermal cell isolation, the protocols are more similar and based on collagen digestion. Once the epidermis is digested or detached, the dermal tissue is incubated with a single [9,11,13,20,22] or a combination [21] of collagenases for various times, varying from 1 [20] to 20 [11] hours at 37 °C. In all cases, these matrix metalloproteinases cleave collagen molecules at a unique Gly-Leu/Ile bond, degrading their triple-helical main structure [27]. Although no significant differences were observed in the fibroblast culture parameters regarding the cell isolation protocol used, new strategies, such as the addition of platelet-rich plasma to the culture medium, could improve and increase fibroblast growth due to its high content of growth factors, such as the vascular endothelial growth factor, fibroblast growth factor, platelet-derived growth factor, transforming growth factor and insulin-like growth factor [28]. These factors have been described to promote wound healing [29] and, moreover, the clinical potential of platelet-rich plasma for cutaneous diseases has also been probed [30].

The higher epithelial cell yield after isolation with the LOEX-Protocol than the UPCIT-Protocol is consistent with previous studies where the application of the two-step digestion protocol allowed for isolating a higher number of epithelial cells/cm^2^ of biopsy (1 × 10^6^–2.6 × 10^6^ cells/cm^2^) [10,22,31] than when a mechanical detachment of the epidermis and subsequent digestion was applied (3.3 × 10^5^–6.6 × 10^5^ cells/cm^2^) [17], which is similar to the UPCIT-Protocol’s value. Interestingly, a higher number of epithelial cells was isolated (2 × 10^6^–5 × 10^6^ cells/cm^2^) when skin biopsies are directly digested without trimming the dermis [10,13,14,15,31], although the risk of fibroblast contamination is higher, due to the unspecific separation, when using trypsin [10,26] and, therefore, an increased percentage of vimentin-positive cells (fibroblasts) has been reported after various times in culture [10]. Among the two-step digestion protocols, when thermolysin is applied for the first digestion, the epithelial cell yield is higher than using other enzymes, such as dispase or trypsin [8,31], at the same step.

The colony-forming efficiency and total number of colonies were similar in both protocols, although slightly higher using the two-step digestion protocol (LOEX-Protocol) compared to the one-step protocol (UPCIT-Protocol) at P0. Similar results have been reported previously [10,32,33]. This is likely due to a better detachment of the dermis from the epidermis at the basement membrane level by thermolysin [26,33], which decreases the incubation time with trypsin, and, therefore, epithelial cell viability is less affected [15].

In addition, the total number of colonies was increased from P0 to P1 in both protocols, probably due to the keratinocytes’ culture conditions (specific medium and feeder layer), which allow a good proliferation of keratinocytes, through maintaining Sp1 expression, and reduce the growth potential of the residual fibroblasts [10,34]. A lower proportion of holoclones was observed in P1 compared to P0 for both protocols. This is attributed to the fact that the cells grew faster in P1 and the culture was stopped earlier (at day 7–10 for P1 and day 10–14 for P0) because the colonies were close to each other. Therefore, these results have to be confirmed in future experiments. 

Finally, culturing keratinocytes through passages can cause stress and reduce the number of epithelial stem cells [35]. Therefore, the culture conditions should be controlled since a decrease in stem cells could have a negative impact on human tissue-engineered skin substitute manufacturing.

The clinical implications of these results are that, regardless of the skin cell isolation methodology applied, only a minor significant difference was observed in terms of epithelial cell yield just after isolation. However, during the cell culture of keratinocytes and fibroblasts, similar results demonstrate that both protocols are robust and comparable and allow for obtaining a sufficient number of cells, in an appropriate period of time, to be able to manufacture human bilayered tissue-engineered skin substitutes for clinical applications as fast as possible. Therefore, this information could be useful for clinical laboratories with the same purposes since they could adapt their own protocols or use these as a reference to develop and optimize their preclinical and clinical research. 

## 4. Materials and Methods

### 4.1. Cell Isolation and Culture

Human keratinocytes and fibroblasts were extracted from three human adult skin biopsies (abdomen (n = 2) and face (n = 1)) from healthy women (49, 49 and 60 years old). Before starting LOEX and UPCIT isolation protocols, adipose tissue and/or blood traces were removed.

#### 4.1.1. Two-Step Digestion Protocol (LOEX-Protocol) 

Human skin samples were washed in 30 mL of a solution constituted of PBS 1X, 100 U/mL penicillin (Sigma-Aldrich, Saint Louis, MO, USA), 25 μg/mL gentamicin (Gemini Bio, West Sacramento, CA, USA) and 0.5 µg/mL fungizone (Bristol-Myers Squibb, New York City, NY, USA) by shaking for 2 min at room temperature (10 times). Then, human skin biopsies were transferred to a 60 × 15 mm Petri dish (Fisherbrand^®^, Waltham, MA, USA) with the epidermis facing up and cut into several pieces 1–2 mm wide. Tissues were digested for 16 h at 4 °C in 10 mL of a solution composed of 0.5 mg/mL thermolysin (Sigma-Aldrich) dissolved in HEPES 1X (MP Biomedicals, Santa Ana, CA, USA) and 1 mM CaCl_2_ (Sigma-Aldrich) (pH 7.45). Next day, the epidermis and dermis were separated using tweezers, kept in washing solution and prepared for the second enzymatic digestion [9,10].

##### Epithelial Cell Isolation

Fragments of the epithelium were incubated for 25 min at 37 °C in 20 mL of a 0.05% Trypsin (Gibco^TM^, Waltham, MA, USA)/0.01% EDTA stirring solution (J.T. Baker, Phillipsburg, NJ, USA). Then, cell suspension was neutralized, filtered using a Cell Strainer of 100 µm (Fisherbrand^®^) and centrifuged (10 min/24 °C/300× *g*). Finally, epithelial cells were counted, seeded at 40,000 cells/cm^2^ on a feeder layer of irradiated human fibroblasts (8000 cells/cm^2^) and cultured in keratinocyte medium (Dulbecco–Vogt modified Eagle medium (Gibco^TM^): Ham’s F12 (Gibco^TM^), ratio 3:1, supplemented with 24.25 μg/mL adenine (Sigma-Aldrich), 5 μg/mL insulin (Sigma-Aldrich), 0.4 μg/mL hydrocortisone (Galenova, Saint-Hyacinthe, QC, Canada), 0.212 μg/mL isoproterenol hydrochloride (Sigma-Aldrich), 5% bovine HyClone FetalClone II serum (GE Healthcare, Chicago, IL, USA), 10 ng/mL human epidermal growth factor (Austral Biologicals, San Ramon, CA, USA), 100 U/mL penicillin (Sigma-Aldrich) and 25 μg/mL gentamicin (Gemini Bio)) [9,10]. This primary culture was named passage 0 (P0). Including the thermolysin digestion step, the estimated time and cost (considering only the price of the enzymes) for the epithelial cell isolation were 18 h and USD 21.00, respectively. 

##### Dermal Cell Isolation

Dermal tissue was digested for 3 h at 37 °C by stirring in 20 mL of a solution constituted of 0.125 U/mL Type H collagenase (Roche, Basilea, Switzerland) diluted in fibroblast medium (Dulbecco–Vogt modified Eagle medium (Gibco^TM^) supplemented with 10% Avantor Seradigm FB Essence (Avantor^®^, Radnor, PA, USA), 100 U/mL penicillin (Sigma-Aldrich) and 25 μg/mL gentamicin (Gemini Bio)). After incubation, cell suspension was filtered using a Cell Strainer of 100 µm (Fisherbrand^®^), separated into four 50 mL tubes (5 mL/tube) and digestion was neutralized with 45 mL/tube of fibroblast medium. After centrifugation of the cell suspensions (10 min/24 °C/450× *g*), the supernatant was discarded until 10 mL were left in each tube; pellets were resuspended and combined to have two tubes with 20 mL in each. These were centrifuged (10 min/24 °C/450× *g*) and previous steps were repeated once to obtain one tube with 20 mL of cell suspension. Finally, this tube was centrifuged (10 min/24 °C/450× *g*). Dermal cells were counted and seeded at 40,000 cells/cm^2^ in fibroblast medium [9,10]. This primary culture was named passage 0 (P0). Including the thermolysin digestion step, the estimated time and cost (considering only the price of the enzymes) for the dermal cell isolation were 21 h and USD 19.50, respectively. 

#### 4.1.2. One-Step Digestion Protocol (UPCIT-Protocol)

Firstly, human skin biopsies were washed for 30 min, submerged in a solution constituted of PBS 1X, 100 U/mL penicillin (Sigma-Aldrich), 25 μg/mL gentamicin (Gemini Bio) and 0.5 µg/mL fungizone (Bristol-Myers Squibb) at room temperature. Then, the samples were transferred to a 60 × 15 mm Petri dish (Fisherbrand^®^) with the epidermis facing down. Most of the dermis was mechanically detached using scissors and a scalpel and kept for fibroblast isolation [11]. 

##### Epithelial Cell Isolation

Epidermis was mechanically fragmented into small pieces using scissors and incubated for 15 min in a stirring 5 mL of TrypLE Select Enzyme 10X (Gibco^TM^) at 37 °C (8 cycles). After each cycle, digested solution containing the cells and pieces of tissue was filtered using a Cell Strainer of 100 µm (Fisherbrand^®^). The filtered cell suspension was neutralized using 10 mL of keratinocyte medium and kept while remaining tissue was digested again. After the 8th cycle, the cell suspension was centrifuged (10 min/24 °C/300× *g*) [11]. Cells were counted and seeded at 130,000 cells/cm^2^ on a feeder layer of irradiated human fibroblasts (8000 cells/cm^2^) and cultured in keratinocyte medium. This primary culture was named passage 0 (P0). The estimated time and cost (considering only the price of the enzymes) for the epithelial cell isolation were 5 h and USD 81.00, respectively.

##### Dermal Cell Isolation

Dermis was fragmented using scissors and digested using a stirring solution of 2 mg/mL of Type I collagenase (Gibco^TM^) in Dulbecco–Vogt modified Eagle medium (Gibco^TM^) for 20 h at 37 °C. The digested tissue was filtered using a Cell Strainer of 100 µm (Fisherbrand^®^), neutralized using fibroblast medium and centrifuged (20 min/24 °C/450× *g*) [11]. Finally, dermal cells were counted and seeded at 115,000 cells/cm^2^ in fibroblast medium. This primary culture was named passage 0 (P0). The estimated time and cost (considering only the price of the enzymes) for the dermal cell isolation were 22 h and USD 11.00, respectively.

#### 4.1.3. Culture Maintenance and Passages

The culture medium of keratinocytes and fibroblasts was changed every two days until cells reached 90–95% confluence; then, cells were trypsinized and cultured again until they reached P4. From P1 to P4, cell seeding density in both protocols was the same for keratinocytes (6500 cells/cm^2^) and fibroblasts (8000 cells/cm^2^).

### 4.2. Cell Size, Viability and Yield

The number of human epithelial and dermal cells and their diameter (Ø) were measured using a Z2 Coulter^®^ Particle Count and Size Analyzer (Beckman Coulter^TM^, Brea, CA, USA). Cell size was evaluated just after isolation (Fresh Cells) and at the end of each passage from P0 to P4. Setting parameters were established between 7 µm and 21 µm for fresh cells and between 8 µm and 24 µm for cells cultured from P0 to P4. The mean size value was used for statistical analysis.

Cell viability was determined by trypan blue staining (Gibco^TM^). The viable cell yield per cm^2^ was calculated using the total number of cells and the area of each human skin sample for fresh cells, and the surface area of the culture flasks for cells recovered from P0 to P4. 

### 4.3. Culture and Population Doubling Time

For each passage, the population doubling time was calculated by dividing the number of doublings (n) by the number of days (culture time). The number of doublings (n) over the culture period was calculated using the following mathematical equation, where the total number of cells at the end of the culture process (Ne) and the initial number of cells seeded (Ni) were considered:

$$n=\frac{\log Ne-\log Ni}{\log2}$$

### 4.4. Clonogenicity of Epithelial Cell Cultures

The size (diameter, Ø) and the number of colonies were evaluated for epithelial cells seeded at P0 and P1. For a given biopsy, passage and isolation protocol, 5 culture flasks (T25 cm^2^) were seeded with 1000 epithelial cells (keratinocytes)/flask and cultured for the same number of days for both protocols. Keratinocyte medium was not changed during the first 5 days of culture, and then medium was changed every two days until colonies started to join with each other.

Colonies were washed 3 times with 4 mL of PBS 1X at 37 °C and fixed with 4 mL of filtered formaldehyde (3.7%) (Chaptec, Montréal-Est, QC, Canada) for 30 min at room temperature. Then, colonies were stained with 1.5 mL of Rhodamine 1% (Thermo Fisher Scientific, Waltham, MA, USA) for 15–20 min at room temperature, rinsed with distilled water and dried.

Colonies were manually counted and classified by their diameter size, measured using ImageJ software (v1.53e, NIH, Bethesda, MD, USA), into i) holoclones: Ø ≥ 4.5 mm, formed by stem cells that rapidly grow; ii) paraclones: Ø ≤ 1.5 mm, constituted by cells programmed for limited growth, which consequently form uniformly small, terminal colonies; and iii) meroclones: 4.5 mm > Ø > 1.5 mm, containing cells with different proliferative capacities [36].

Using this data, CFE [37] and the percentage of each type of colony, considering the total number of colonies, were calculated:

$$CFE=\frac{Number\;of\;holoclones}{Initial\;number\;of\;keratinocytes\;seeded\;(1000)}\times 100$$

$$\%\;of\;Total\;Colonies=\frac{Number\;of\;colonies\;(for\;each\;type)}{Total\;number\;of\;colonies}\times 100$$

### 4.5. Keratin 19 (K19) Analysis by Flow Cytometry

The number of K19-positive keratinocytes after P0 and P1 was evaluated by flow cytometry. Cells were recovered after trypsinization, washed in PBS 1X (3 times) and fixed in filtered formaldehyde 3.7% (pH = 7) (Chaptec) for 30 min at 4 °C (1 mL of formaldehyde /10^6^ cells). Then, cells were washed again with cold PBS 1X (3 times) and kept at 4 °C in 1 mL of PBS 1X/10^6^ cells until flow cytometry analysis.

For each protocol and passage, two samples of at least 400,000 cells were immunolabeled. Cells were centrifuged at 450× *g* for 5 min at 15 °C. The supernatant was discarded and keratinocytes were washed with 500 µL of 10% BD Perm/Wash Buffer (BD Biosciences, Franklin Lakes, NJ, USA) diluted in MilliQ water. Then, cells were immunolabeled for 45 min at room temperature in darkness with mouse IgG2a antihuman K19 primary antibody (clone A53-B/A244, gift from U. Karsten, Institute of Biological Sciences, University of Rostock, Germany; 1:200). For negative controls, keratinocytes were incubated with mouse IgG2a antibody (DAKO, Glostrup, Denmark; 1:20). After 3 washes, samples were immunolabeled with PE-conjugated goat antimouse IgG2a secondary antibody (Invitrogen, Waltham, MA, USA; 1:200) and incubated for 45–60 min at room temperature in darkness. After 3 washes, each sample was resuspended in 200 μL of PBS FACS solution (PBS 1X, 0.5 mM EDTA (Sigma-Aldrich) and 2% Serum) and analyzed using BD FACSMelody™ Cell Sorter (BD Biosciences).

### 4.6. Statistical Analysis

Microsoft Excel software (v2308) was used for primary analysis of raw data, and GraphPad Prism 8 (San Diego, CA, USA) and FlowJoTM v10 (BD Biosciences) were used for statistical analysis and graphs. Normal distribution of the data was corroborated by Shapiro–Wilk test. Then, depending on the comparison, one-way ANOVA and Tukey’s multiple comparisons tests, or multiple *t*-tests and Holm–Sidak’s method, were used to analyze the results. All values were presented as mean value ± standard deviation (SD). The significance threshold was set at 0.05.

### 4.7. Ethics 

This study was conducted according to the Declaration of Helsinki and was approved by the research ethics committee for human subjects of the CHU de Quebec-Université Laval. All tissue donors provided informed written consent. 

## 5. Conclusions

This comparative in vitro study demonstrated that the application of the two-step digestion protocol (LOEX) or one-step digestion protocol (UPCIT) does not differentially affect the isolation and culture of human dermal fibroblasts. However, the use of the LOEX-Protocol increased the viability and cell yield of the skin epithelial cells after the isolation process, but no differences were observed once the keratinocytes were cultured through different passages. Therefore, both isolation protocols are useful for human skin cell isolation and their subsequent use for the clinical fabrication of human bilayered tissue-engineered skin substitutes. 

## Data Availability

All data generated or analyzed during this study are included in this published article.

## Abbreviations

Ø: diameter; CFE: colony-forming efficiency; K19: Keratin 19; LOEX: tissue engineering lab, a research center recognized by Université Laval; P: passage; TESS: tissue-engineered skin substitute; UPCIT: Unidad de Producción Celular e Ingeniería Tisular.
